# Molecular Insights into Nutrient-Driven Regulation of Trophic Strategies and Life-Cycle Shifts in Bloom-Forming Mixotrophic Dinoflagellates

**DOI:** 10.3390/ijms27198622

**Published:** 2026-09-26

**Authors:** Sergei O. Skarlato, Mariia A. Berdieva, Vera O. Kalinina, Hendrik Schubert, Irena V. Telesh

**Affiliations:** 1 Institute of Cytology of the Russian Academy of Sciences, St. Petersburg 194064, Russia; sergei.skarlato@mail.ru (S.O.S.); maria.berd4@yandex.ru (M.A.B.); verakamakalinina@gmail.com (V.O.K.); 2 Institute for Biosciences, Faculty of Mathematics and Natural Sciences, University of Rostock, 18051 Rostock, Germany; 3 Zoological Institute of the Russian Academy of Sciences, St. Petersburg 199034, Russia

**Keywords:** dinoflagellates, harmful algal blooms, kleptoplasty, life cycle, mathematical modeling, mixotrophy, molecular ecology, transcriptomic analysis

## Abstract

Research into the molecular machinery of eukaryotic unicellular organisms (protists) offers a unique opportunity to unveil the mechanisms of intricate cellular processes and complete life cycles at the level of a single cell operating as a whole organism. Knowledge of protistan molecular biology and life-cycle shifts as adaptations to varying physical–chemical features of the environment helps resolve important issues like evaluation of the balance between external abiotic triggers and internal molecular drivers of cell development. Understanding the molecular mechanisms of these processes is crucial for prognostic modeling of the population growth of ecologically and economically important protists, like toxic species that form harmful algal blooms (HABs) that currently expand worldwide. Here, we review the basic features of bloom-forming mixotrophic dinoflagellates as drivers of their excess proliferation and HABs. We unveil key components of molecular machinery involved in life-cycle regulation of dinoflagellates and highlight the deficiency/redundancy of nitrogen- and phosphorus-containing substrates as modulators of life cycles, diversity and nutrition strategies, including mixotrophy and kleptoplasty. We discuss linkages between cellular and molecular biology, omics and ecological modeling, since merging the research approaches of these disciplines is a promising perspective for future progress of molecular biology within the framework of translational aquatic ecology.

## 1. Introduction

Molecular machinery, including mechanisms maintaining signal transduction and gene regulation networks, provides a solid though flexible basis for the development of cells and organisms within the entire biological life hierarchy, ranging from a variety of intracellular processes to complete life cycles. Among all living creatures, eukaryotic unicellular organisms (protists) are of particular importance for molecular ecologists. They provide a unique opportunity to study these mechanisms at the level of a single cell functioning as a whole organism. Knowledge of protistan molecular biology, including life-cycle shifts as adaptations to varying physical and chemical features of the environment, is of paramount significance. This information helps resolve sophisticated issues such as chaotic population dynamics [1,2]. It also promotes the evaluation of the balance between external triggers (abiotic characteristics) and internal (cellular and molecular) drivers of cell and organism development [3,4]. A comprehensive understanding of molecular mechanisms of these processes is crucial for the accurate assessments and forecasts of population growth of the ecologically and economically important protists. Of particular interest are toxic and potentially toxic microalgae that cause harmful algal blooms (HABs).

Nowadays, HABs are intensifying and expanding not only at the regional level, e.g., as a result of excess nutrients from aquaculture development and increased discharges of agricultural wastewaters and fertilizers [5,6], but also worldwide [7,8,9,10,11]. They occur in a variety of marine and continental water basins of different types, from small eutrophic ponds to vast oligotrophic oceanic regions, since many of these areas are strongly affected by climate change and anthropogenic eutrophication [12,13,14]. The devastating impacts of HABs on aquatic ecosystems and their biota are well known and have been repeatedly reviewed elsewhere [8,9,13,15,16,17,18,19]. In brief, usually by the end of a HAB, the decomposition of algal biomass accumulations results in oxygen depletion in the water column and deterioration of the quality of natural waters. The latter effect destabilizes the environment and causes irreparable harm to the flora and fauna, fisheries and fish farming, aquaculture, tourism and public health. These issues, along with the direct toxic effects of some bloom-forming protists on aquatic organisms and humans, are generally listed among the major negative consequences of HABs [14,20,21]. However, the bloom onset most often remains enigmatic and unpredictable because of a number of impartial reasons. On the one hand, it depends on numerous, variable and mutually dependent environmental factors. On the other hand, the contribution of molecular data on bloom-forming species in disclosing the triggers and drivers of emergence, development, timing, magnitude and decay of HABs is largely unappreciated. A lack of this knowledge is a major reason for the insufficient effectiveness of many current prognostic models of HABs.

One of the key HAB-forming groups of protists are dinoflagellates (Dinoflagellata). Numerous studies prove that dinoflagellates play a principal role in microplankton biodiversity [22,23,24,25,26,27]. A significant portion of the ca. 2500 known morphological species of these microalgae causes massive population outbreaks, or blooms (also called “red tides”), mainly in coastal waters. Moreover, out of the 200 reliably known morphological taxa of various marine phytoplankton whose cells produce toxins, more than 105 are dinoflagellates [6]. Their toxins and other secondary metabolites cause up to 500,000 human intoxications per year, with an average mortality rate of approximately 1.5% [28].

The effective bloom-forming capacities of many dinoflagellates are backed up by their molecular, cellular, physiological and metabolic peculiarities [23,27,29,30]. One of these particular features is their capacity for mixotrophic nutrition and kleptoplasty [31]. This capacity enhances cell population development, often leading to HABs [18,19,32,33]. However, little numerical data on the contribution of these specific characteristics to dinoflagellate HAB formation are available so far. Moreover, mathematical models of these devastating events, based on computational approaches, transcriptomic data, laboratory and theoretical experiments and the analyses of long-term data series, as well as generalizations of the available knowledge, are scarce [34]. Meanwhile, if available, it is possible that they have significantly contributed to the development of comprehensive HAB control strategies for environmental management aimed at effective protection of natural aquatic ecosystems and their resources.

Thus, although many field observations exist for HABs, the central knowledge gap is that molecular evidence (transcriptomic signatures, life-cycle transition, kleptoplasty, etc.) has rarely been integrated into HAB predictive models. In this article, we aimed to fill in the above gap by presenting an overview of nutrient-driven regulation of trophic strategies and life-cycle shifts in bloom-forming dinoflagellates and assessing available model integrations of these data. Here, molecular features of mixotrophic dinoflagellate cells are considered as the basis for their enhanced proliferation, including the extreme red-tide events. We discuss the deficiency and redundancy (excess) of nutrition substrates containing nitrogen (N) and phosphorus (P) and their roles in dinoflagellate population growth and diversity, studied via experiments as well as in nature. We also highlight the effects of variations in nutrient concentrations and availability as modulators of life cycles and nutrition strategies (including mixotrophy and kleptoplasty) of HAB-forming planktonic dinoflagellates. In some sections, the article focuses on the biology of a model red-tide microalgae species: the globally distributed mixotrophic, potentially toxic armored dinoflagellate *Prorocentrum cordatum* (Ostenfeld) [35], also known by its main synonym, *Prorocentrum minimum* (Pavillard) [36]. Here, we review its peculiar molecular features, studied in the experiments with laboratory cultures. We also summarize the data on cellular and molecular mechanisms that drive the proliferation of natural *P. cordatum* populations forming blooms and characterize some of their traits in the southwestern coastal waters of the Baltic Sea.

Special attention is paid to the results of the transcriptomic analyses of molecular machinery involved in dinoflagellate life-cycle regulation and the molecular basis of their mixotrophic strategies in a changing environment. In conclusion, we highlight the dire necessity of including molecular data on bloom-forming mixotrophic dinoflagellates in conceptual and mathematical ecological models for proper evaluation and forecasting of HABs. This approach is mainstream in translational ecology, which defines a collaborative effort among scientists and stakeholders to rapidly translate environmental problems into action [37]. In the current study, translational aquatic ecology (TAE) is referred to as a recently emerged scientific discipline that capitalizes on smart conversion of the fundamental findings in aquatic ecology and hotspot molecular biology discoveries conducive to their practical use in essential human life activities [38]. This concept is of particular relevance in a fisheries management context as well as in biotechnology, aquaculture and environmental management [37,38]. The authors believe that the review could help strengthen the linkages between cellular and molecular biology, omics, aquatic ecology and ecological modeling. Bridging the knowledge–action gap concerning dinoflagellate HABs, their molecular drivers, ecological consequences and nature protection measures by merging the approaches of the above disciplines is considered a promising perspective for the future studies that will secure the progress of molecular ecology within the TAE framework.

## 2. Major Roles of Dinoflagellates in Aquatic Ecosystems

### 2.1. General Characteristics, Biodiversity and Taxonomy

Dinoflagellates form an important marine and brackish-water phytoplankton cluster characterized by pronounced taxonomic and functional diversity. They are crucial to aquatic ecosystems due to their high abundances, biomass and productivity. Dinoflagellates effectively fulfill nutrient cycling and energy transfer, synthesize specific toxins, implement benthic–pelagic coupling, form harmful red tides and thus have major ecological significance [18,23,27,39,40,41,42]. The maximum taxonomic diversity of dinoflagellates and their highest abundances are usually registered in marine coastal waters and river mouths [43,44].

The most peculiar features of dinoflagellate cell organization comprise the complexity of their cellular coverings [45,46,47], unusual structure of the nucleus and permanently condensed chromosomes with distinct chromatin architecture [48,49,50]. They are also characterized by variability and specific organization of ion channels that shed light on the cell physiology [51,52,53] and extraordinary diversity of plastids [54,55]. The molecular features of dinoflagellates involve one of the largest genomes among eukaryotes, extensive gene duplication, peculiar gene organization patterns such as tandem arrays, specific features of splicing, predominance of post-transcriptional and translational regulation over transcriptional control and other processes [56,57,58,59,60].

Based primarily on molecular–phylogenetic studies of dinoflagellate cell biology, modern taxonomy and systematics attribute these protists to a large monophyletic group, Alveolata, along with ciliates (Ciliophora) and sporozoa (Apicomplexa) [61,62,63]. Molecular phylogeny proves that dinoflagellates are most closely related to Apicomplexa but not to Ciliophora [64]. Meanwhile, the evolutionary line of Alveolata belongs to a supergroup SAR, along with Stramenopila and Rhizaria [65]. The latter supergroup was rather recently expanded to TSAR due to the addition of the phylum Telonemia after new molecular phylogeny data were obtained [66].

### 2.2. Functional Traits and Secondary Metabolite Production

Among functional traits, the ability of dinoflagellates to form symbiotic relations with corals is well known [67]. In marine and coastal ecosystems, these protists are crucial, being major primary producers along with diatoms [68]. Dinoflagellates are an effective biological “carbon pump” and a means of transporting nutrients within the food web, serving as food for microzooplankton and fish larvae [40,69,70]. They can form HABs in marine and brackish-water coastal areas worldwide [14,36,71,72], including the arctic seas [23,73].

Many dinoflagellates produce structurally diverse toxins that are derived via divergent pathways [74,75,76,77]. The main toxin-producing dinoflagellate species in plankton belong to the genera *Prorocentrum*, *Alexandrium*, *Dinophysis*, *Gambierdiscus*, *Ostreopsis* and *Karenia*. The dinoflagellate toxins cause nausea, vomiting, abdominal pain, diarrhea and neurologic symptoms such as fatigue, myalgias, pruritus, circumoral paresthesia, reversal of hot and cold sensations, psychiatric abnormalities and memory loss; the neurologic symptoms may persist for months to years [28].

Along with toxins sensu lato, marine dinoflagellates also produce a wide range of other biologically active secondary metabolites, including unique pigments like peridinin [78] and specific proteins, although the roles of some of them remain poorly understood [35,79,80]. As demonstrated for various microalgae, some of these compounds possess antiviral [81], antibacterial [82], antifungal [83] and antitumor [84] properties; therefore, they can be used in the pharmacology and food industries. Much attention has been paid to dinoflagellate metabolites containing complex compounds with diverse chemical natures [85], including macrolides, cyclic polyethers, spirolides, purine alkaloids and other components [86].

Currently, dinoflagellates are being actively used in the search for new natural migrastatic substances that could inhibit cancer cell motility, thereby preventing metastasis. For example, recent studies using the MALDI-TOF technique [87] have identified biologically active secondary metabolites, including the pigments chlorophyll *a* and peridinin, and proteins (chaperones, cytoprotective and antioxidant proteins), produced by the potentially toxic dinoflagellate *Prorocentrum cordatum* [80,88]. Specifically, the latter studies used adherent murine hepatoma MH22a cells as one of the target models. An ethanol extract of *P. cordatum*, due to its low toxicity and ability to suppress cell adhesion and migration, appeared to be the most promising potential biologically active agent [80].

### 2.3. Life Cycle and Benthic–Pelagic Coupling

Dinoflagellates (except *Noctiluca* spp.) have a complex haplontic life cycle. In general, in favorable conditions, haploid vegetative cells (n) actively proliferate by mitosis. Under certain triggers, cells can enter the sexual stage of the life cycle and differentiate into gametes. The gametes fuse to produce motile zygotes (2n), also called planozygotes, which typically bear two longitudinal flagella. Planozygotes can encyst and form sexual dormant, long-term, thick-walled resting cysts (hypnozygotes, 2n)—or, alternatively, differentiate into planomeiocytes directly—and undergo meiosis, producing a new vegetative generation (n). Vegetative cells can also form resting cysts (n) to survive unfavorable environmental conditions [39,89]. Another type of dinoflagellate cyst is the temporary cyst—also referred to as a pellicle cyst, ecdysal cyst, or thecate cyst—a thin-walled, short-lived, non-motile, non-dormant stage formed by a motile cell (vegetative or sexual) in response to unfavorable conditions, such as mechanical, salinity or cold stress or bacterial invasion [90,91,92]. For some species, short-term encystment of planozygotes has also been shown [93].

The sexual process in the life cycle of dinoflagellates is known and generally regarded as both a survival strategy in adverse environmental conditions, like nutrient deficiency, stressful temperature and salinity regimes, intense grazing or parasite infection [94,95,96], and a proliferation strategy [97].

In aquatic ecosystems, dinoflagellates also facilitate connectivity between the pelagic and benthic communities. Their cysts accumulate at the bottom but re-inoculate the water column when favorable conditions for excystation are restored [38,39,98,99,100]. The life-cycle complexity of these protists defines the variability of their dormancy strategies. The long-term resting cysts are characterized by a significant period of dormancy, which can reach several months or even years; the temporary cysts stay immotile for a few hours or days, although sometimes for months [39,91,101].

Consequently, the nature of the benthic–pelagic coupling involving HAB-forming dinoflagellates is complex and ambiguous. These interactions play a dual role in the formation of red tides: first, due to cyst formation and deposition at the bottom, and second, because of the composite, nonlinear effects of benthic feeding on the nutrient content in water. Concerning the latter, on the one hand, the excretion of available nutrients by organisms in benthos such as bivalves, accompanied by the excystation of dinoflagellates under favorable conditions and their release into plankton, increases the trophic status of a water body and promotes HABs. On the other hand, the elimination of microalgae by these same mollusks during their intense feeding by filtration reduces the population size of bloom-forming species and thus prevents red tides [18].

### 2.4. Ecological Niches and Intra-Population Heterogeneity

Many HAB-forming planktonic dinoflagellates thrive in various aquatic environments and successfully withstand external stressors. This is to great extent due to their wide ecological niches, particularly with respect to temperature, pH, salinity and the concentration of basic nutrients; a good example is a cosmopolitan marine mixotrophic species *Prorocentrum cordatum* [102]. Additionally, largely due to the mixotrophy of many dinoflagellates [103,104,105,106], their broad nutrition spectrum is another great ecological advantage of these protists [107,108]. Mixotrophic dinoflagellates utilize a wide range of organic and inorganic resources available for their growth and reproduction, often changing trophic preferences depending on environmental conditions [11,109]. Specifically, by using stable isotope tracers and NanoSIMS, it was shown that individual *P. cordatum* cells within a population displayed significant heterogeneity in the rates of nutrient uptake and extent of urea-mediated inhibition (by 30–40%) of nitrate uptake [110]. Thus, by starting to consume urea at a high rate as soon as it appeared in the environment, *P. cordatum* demonstrated rates of urea-N uptake that were 1.6–4.1 times higher than those of nitrate-N uptake [110]. Moreover, these dinoflagellates also demonstrated the uncoupled assimilation of carbon and nitrogen from urea and the amino acid glycine [111]. The discovered functional heterogeneity of these protist populations, leading to the putative co-existence of various sub-populations with differing nutrition modes, ensures the ecological success of these dinoflagellates and enhances their competitiveness in stressful environments. These advantages can also serve as a functional basis for enhanced proliferation causing red tides under the impact of even sporadic urea inputs to coastal ecosystems. However, the direct empirical data proving that intra-population heterogeneity of dinoflagellates leads to HABs is lacking.

### 2.5. Competitive Advantages, Blooms and Range Expansion

The above features contribute to the proliferation of dinoflagellates in diverse habitats, in water bodies of different types and to the expansion of red-tide zones therein (Figure 1). They also allow these protists to compete successfully with closely related species, as demonstrated by the invasion history of *P. cordatum* in the Baltic Sea and its displacement of the previously dominant congeners among planktonic dinoflagellates therein [102]. Thus, knowledge of the nutrition strategies of these flagellates and new data on their ecological niches have made a significant contribution to HAB research [112]. Specifically, it was shown that different trophic strategies of these microorganisms determine their ability to rapidly proliferate by modulating the growth rate and reproductive efficiency, providing competitive success, high invasion potential and range expansion [18,33,42,102,110,113,114].

Enumeration of peculiar molecular, cellular and physiological features and functional traits of dinoflagellates can be continued, and their list can be much longer. However, we will cease it for now, since the subsequent sections of this review will focus primarily on the essential nutrients and their roles in the molecular machinery of dinoflagellate trophic strategies, reproduction and life-cycle regulation. All together, these characteristics secure the pronounced adaptability of these protists to climate changes and anthropogenic stresses and enhance their persistence, population growth and HAB formation.

## 3. Nitrogen and Phosphorus as Essential Nutrients Modulating Dinoflagellate Cell Physiology

### 3.1. Nitrogen

Nitrogen (N) and phosphorus (P) are the two biogenic elements whose availability in the environment constitutes one of the principal prerequisites regulating various aspects of dinoflagellate cell physiology. Nitrogen is a structural component of amino acids, nucleotides and chlorophyll molecules. The availability of nitrogen limits the productivity of marine microbial communities and plays an essential role in structuring phytoplankton species composition [115,116,117]. Marine phytoplankton provide a major proportion of global primary production and modulate biogeochemical cycles by assimilating nitrogen. It is usually assumed that most microorganisms can use dissolved inorganic nitrogen (DIN) in the form of nitrate, nitrite and ammonium [118]. Dissolved organic nitrogen (DON) can be a large pool in the oceans and an even larger one in coastal waters. A significant fraction of this pool is composed of amides (including urea); it also includes heterocyclic compounds, proteins and amino acids [118]. Nitrogen limitation affects metabolic processes in dinoflagellate cells, enhancing glycolysis, the pentose-phosphate pathway, the tricarboxylic acid cycle and fatty acid metabolism and altering the expression of genes encoding photosystem proteins [119,120,121,122,123]. Nitrogen deficiency activates nitrate/nitrite and urea transporters to increase assimilation of available nitrogen sources [121,122].

### 3.2. Phosphorus

Phosphorus is a biogenic element that plays a crucial role in cells, as it is a component of nucleic acids (DNA and RNA), cell membranes (phospholipids), signaling molecules (inositol triphosphate and cAMP), nicotinamide adenine dinucleotide (NAD) and its phosphorylated form (NADP), adenosine triphosphate (ATP, an energy currency), etc. Phosphorylation activates many enzymes necessary for maintaining basic metabolic pathways and regulating the cell cycle. Thus, phosphorus availability has a significant impact on the viability and growth of marine phytoplankton and, consequently, on their primary production in the seas and oceans.

Phosphorus can be available to dinoflagellates in the form of dissolved inorganic phosphate, DIP (primarily orthophosphate [Pi]) and as a variety of dissolved organophosphorus (DOP) compounds such as ATP, cytidine-5-monophosphate, fructose-6-phosphate, glucose-6-phosphate, glycerophosphate, uridine-5-monophosphate, phenyl phosphate, RNA, etc. [124,125,126,127,128]. DOP appears to be the main source of this nutrient during algal blooms, when inorganic nutrients are depleted [129]. Phosphorus deficiency stimulates phosphate uptake and intracellular recycling [130,131,132,133]. For example, proteomic analysis of phosphate-starved *Karenia mikimotoi* revealed the overexpression of vacuolar phosphate transporters, proteins associated with vesicular transport and proteins responsible for lipid and phospholipid degradation [130]. *K. mikimotoi* and *Prorocentrum shikokuense* Y.Hada (the authors’ names of the species are given when first mentioned in the text only for the species that are not indicated in Supplementary Table S1, except for *Prorocentrum cordatum* and its synonym, *P. minimum*) have also been shown to replace membrane phospholipids partially with glycerol glycolipids and betaine lipids [134,135]. In freshwater ecosystems, despite the historical “dogma” defining them as strictly phosphorus-limited [136], many systems exhibit co-limitation, where both N and P availability simultaneously constrain primary production [137,138].

### 3.3. Effects of Nitrogen and Phosphorus on Toxin Biosynthesis

Notably, toxin biosynthesis depends on the availability of both elements. Primarily, nitrogen is required for the production of saxitoxins, as shown for *Alexandrium tamarense* and *Alexandrium minutum* [139,140]. The most recent studies indicated that high nitrogen supply enhanced not only saxitoxin biosynthesis but also toxin release: excess nitrogen promoted *sxtA4* gene expression by *Alexandrium catenella* (Whedon & Kofoid) Balech, with a positive correlation between *sxtA4* expression and total toxin yield [141]. Nevertheless, the production of okadaic acid (diarrheic shellfish toxin) is modulated by the phosphorus level [142].

## 4. Trophic Strategies of Dinoflagellates in the Changing Environment: Mixotrophy and Kleptoplasty as Its Peculiar Constituent

### 4.1. Mixotrophy

In recent years, researchers have been paying much attention to studying not only the ecological prerequisites of dinoflagellate blooms but also the intracellular drivers and molecular mechanisms that back up their trophic strategies and secure fast population growth, often leading to HABs [30]. Mixotrophy is one of the nutrition modes typical for many bloom-forming dinoflagellates that combines autotrophy due to photosynthesis in the light using inorganic nutrient substances with heterotrophy in the forms of osmotrophy and phagotrophy [103,104,105,106,143,144]. Osmotrophy involves the consumption of dissolved organic compounds by osmosis. Phagotrophy is the ability of cells to engulf, absorb and assimilate solid organic substances, such as microalgae and bacterial cells. Currently, photo-osmo-mixotrophy is considered a generally accepted and widespread nutritional mode of microalgae [145]. At the same time, mixotrophy, which includes phagotrophy, is not ubiquitous; moreover, the modes of use of this nutritional strategy also vary significantly among diverse planktonic protists [145,146].

The combination of the two strategies, photo-osmo-mixotrophy and photo-phago-mixotrophy, provides high adaptability of protists to fluctuations in the composition and abundance of food resources. Consequently, it increases the ecological homogeneity of many unicellular mixotrophic organisms, which provides the basis for their unification within the concept of “mixoplankton” [147,148]. Mixoplankton (i.e., plankton capable of mixotrophy) are generally characterized by high adaptability to fluctuating environments [149,150]. This is primarily due to the ability of mixoplankton to utilize various alternative resources [110,111], as well as their broad ecological niches, including a wide range of food substrates [102,108,114]. As a result, because of the high adaptability and rapid growth of mixoplankton populations, they are often the cause of HABs [6,7,151,152,153].

New findings repeatedly expand the list of dinoflagellate species exhibiting mixotrophic properties [154,155]. For example, recently it has been shown that none of the dominant HAB-forming dinoflagellate species in the coastal waters of the southwestern Baltic Sea is purely autotrophic. Following the classification by Jeong and co-authors [108], it has been established that seven out of ten of these dominants are mixotrophs, while the remaining three species are heterotrophs [42].

The ability to engage in mixotrophy enhances the resilience of dinoflagellates to changing environmental conditions, including shifts in the composition and concentration of nitrogen- and phosphorus-containing compounds or decreased light, which impedes photosynthesis. Specifically, phosphorus deficiency stimulates phagotrophy in some mixotrophic dinoflagellates, such as *Prorocentrum cordatum* and *Tripos* sp. (the latter was described as *Ceratium furca* (Ehrenberg) Claparède & Lachmann) [156,157]. Moreover, phosphorus deficiency increases the expression and activity of cell-surface alkaline phosphatase [158,159,160]. A lack of phosphorus in the environment induces the expression of plasma membrane and vacuolar phosphate transporters as well as proteins associated with vesicular transport [96,130]. Nitrogen depletion can also induce phagotrophy in *P. cordatum* [157] and *Karlodinium veneficum* (D.Ballantine) J.Larsen (described as *Gyrodinium galatheanum* (Baarud) Taylor in [161]), although to a lesser extent than phosphorus. Activation of endocytosis and phagosome-related pathways has also been detected at the transcriptomic level [122].

Mixotrophy involving phagotrophy is observed in all phytoplankton clades, with the exception of diatoms and cyanobacteria [162]. Moreover, nowadays mixotrophy is considered not just a nutritional strategy but also a unique life strategy for dinoflagellates and many other protists [10,108,163]. The environmental triggering of mixotrophy is highly complex and multidimensional (reviewed in [34]). Recent transcriptomic analyses have revealed that mixotrophy is not just the synergistic process of “grazing while photosynthesizing”; rather, prey availability can trigger large-scale transcriptional reprogramming that shifts cellular metabolism toward heterotrophic pathways [34,164,165]. However, some subtle molecular and cellular aspects of mixotrophic metabolism remain poorly understood [166].

### 4.2. Kleptoplasty in Dinoflagellates

Kleptoplasty is one of the less studied though most enigmatic intracellular mechanisms of mixotrophy [167]. The term “kleptoplasty” refers to the processes of acquisition, accumulation and utilization of kleptoplasts, i.e., unassimilated chloroplasts of microalgae absorbed by heterotroph and mixotroph cells during phagotrophic nutrition [32,168,169,170]. Kleptoplasty is observed in a wide range of organisms, not only in unicellular protists, such as dinoflagellates, ciliates and foraminifera [171,172], but also in multicellular invertebrates, for example, sacoglossan sea slugs of the genus *Elysia* [169] and some flatworms [173].

It has been established that kleptoplasts not only persist but also photosynthesize in the host cell, sometimes for a relatively long period, up to several months [174]. For example, in *Nusuttodinium aeruginosum* (F.Stein) Y.Takano & T.Horiguchi, kleptoplasts can be inherited by the daughter cells through five generations and can even be found in the resting cysts [175]. Along with their own plastids, kleptoplasts in mixotrophs serve as additional “reactors of photosynthesis” [19,33]. Eventually, the photosynthesis of kleptoplasts is diminished and finally ceased, and/or the “stolen” plastid is digested [144]. For the majority of photo-phago-mixotrophic protists, however, the question of how great the contribution of functioning kleptoplasts to the overall photosynthesis and cell growth is remains open.

Dinoflagellates obtain kleptoplasts from prey algae such as various representatives of cryptophytes [161], haptophytes [176], chlorophytes [177], prasinophytes [176], raphidophytes [178], diatoms [179,180] and other dinoflagellates [181,182]. The pathway of plastid acquisition can be intricate. *Dinophysis acuminata* Claparède & Lachmann acquires plastids of the *Geminigera*/*Teleaulax* cryptophyte from the captured ciliate *Myrionecta rubra* (Lohmann) Jankowski [183]. As a result, the two outer plastid membranes, the cryptophyte nucleus and the nucleomorph, are lost. At the same time, the nuclear genome of *D. acuminata* contains five plastid genes from multiple algal lineages, only one of which was derived from a cryptophyte, and this implies instability of the photosynthetic system in these dinoflagellates [183]. Meanwhile, the Antarctic Ross Sea dinoflagellate appears to retain its ancestral secondary plastid and obtains temporary kleptoplasts stolen from haptophyte prey partitioning functions between the two plastid systems [184]. Moreover, this dinoflagellate has acquired nuclear genes by horizontal gene transfer (HGT) from outside the kleptoplast lineage; however, these HGT genes encode kleptoplast-targeted proteins. Their expression is not strongly changed by environmental conditions, and many of them are shared with related dinoflagellates with fully integrated plastids. Such a pattern suggests that the protein targeting system had developed before the plastid became permanently fixed [184]. Thus, the fate and physiological role of captured plastids in a mixotrophic dinoflagellate cell remains a topical though understudied issue.

Laboratory experiments showed that, for example, for *Prorocentrum* spp. (*P. micans* Ehrenberg, *P. cordatum*), cryptophyte microalgae are most often the optimum prey and a source of kleptoplasts in terms of cell size and consumption rate [107]. The results of the long-term Baltic Sea database analyses support this outcome by demonstrating the co-existence of *P. cordatum* and *P. micans* with cryptophyte species like *Hemiselmis virescens* Droop or *Teleaulax* sp. in plankton, resulting in strong and statistically significant (*p* < 0.05) positive correlations between the abundance of *Prorocentrum* spp. and cryptophytes (Table 1).

In the meantime, neither the laboratory studies of dinoflagellate feeding rates nor the field data on their co-occurrence with certain prey objects can provide information on the efficiency of kleptoplasty. Additionally, the experimental investigations of this specific and delicate intracellular process are still problematic. Thus, in most cases, the real contribution of kleptoplasty to dinoflagellate cell growth remains unknown. Therefore, this phenomenon has not been taken into account in the majority of available mathematical models of the growth and population dynamics of mixotrophic organisms [70,163,186].

### 4.3. Modeling of Kleptoplasty Efficiency and Kleptoplasty Index

The only exception known so far is a mathematical model of kleptoplasty based on the fundamental J. Monod model of microorganism growth [187], a series of theoretical experiments (“thought experiments”) and a set of differential equations for a simple biological system, which consisted of one mixotrophic protist cell, one inorganic resource and one organic resource [33]. The model was developed using experimental data on the feeding of the dinoflagellate *Prorocentrum cordatum* on the cryptophyte microalga *Teleaulax* sp. [107,108]. The reproduction rates and morphometry of dinoflagellates were taken into account as basic prerequisites [36].

This model study suggested an original kleptoplasty index (*k*), a new ecological indicator reflecting the quantitative contribution of kleptoplasty to HAB formation [33]. The kleptoplasty index *k* is considered as a function of the dimensionless parameters *α* and *σ*. The parameter *α* represents the photosynthetic contribution of kleptoplasts normalized by that of native chloroplasts; the parameter *σ* describes the balance between the autotrophic and heterotrophic feeding modes. For this model, using the experimental prerequisites, we calculated and compared the feasible cells’ division rates in various resource-rich environments. In particular, the model has shown that kleptoplasty of the mixotrophic *P. cordatum* using *Teleaulax* sp. as food can increase the cell division rate by up to 40%, promoting rapid population growth of these dinoflagellates and subsequent algal bloom [33]. The limitations of the model are due to the scarcity of experimental data on growth rates of bloom-forming mixotrophic protists in environments suitable for mixotrophy. Meanwhile, although the current lack of extensive field validation of the kleptoplasty index is a constraint restricting its application, hopefully this can be a prospective task for further research.

In future modeling studies, the assessment of the role of kleptoplasty in the formation of dinoflagellate HABs can be correlated with the major cellular parameters and molecular mechanisms that determine the functioning of these protists. Thus, further research into the molecular basis of kleptoplasty as one of the key mixotrophic nutrition mechanisms affecting dinoflagellate HABs can be a future prospect of molecular ecology, which could help understand algal blooms and contribute to prognostic mathematical modeling of HABs.

## 5. Effects of Nitrogen Redundancy on the Expression of N-Transporter Genes, Transcriptional Responses, Diversity and Dinoflagellates Population Growth

### 5.1. Nitrogen Excess Affects Gene Expression

The abundance, composition and proportions of different inorganic and organic nutrients in the environment play a crucial role in mixoplankton development and proliferation. The presence of different forms of nitrogen is mandatory for various cellular metabolic processes of dinoflagellate cells that secure their maintenance, growth and reproduction (see also Section 6). For example, experimental results suggest that mixotrophic dinoflagellates *Prorocentrum cordatum* are sensitive to the proportions of N-containing substrates and can adopt the most effective strategy for assimilation of available nitrogen sources in the changing environment, demonstrating a response to shifts in their composition [109,110,111,188]. It was discovered that the expression levels of the genes *dur3* and *nrt2* encoding the transporters of urea and nitrate, respectively, in NO_3_-grown cultures depended on the added nitrogen sources being the lowest in the presence of ammonium [188].

Studies of the transcriptional responses of *P. cordatum* to available N-containing substrates allowed for estimating the influence of NO_3_^−^, NH_4_^+^ and urea on the transcription levels, as well as on the expression of urea and nitrate transporter genes in these dinoflagellates. Specifically, it was shown that *dur3* expression levels were downregulated after the supplementation of additional N sources and were 1.7–2.6-fold lower than in the nitrate-grown culture, while the *nrt2* expression levels decreased 1.9-fold in the presence of NH_4_^+^ [188]. Total RNA and DNA synthesis rates, estimated by the analysis of incorporation of ^3^H-thymidine and ^3^H-uridine in batch and continuous cultures, have demonstrated that the addition of N compounds did not affect the DNA synthesis rates. Meanwhile, the transcription levels increased up to 12.5-fold after excessive N supplementation [188]. These investigations unveiled the role of various nitrogen sources as biomarkers of dinoflagellate proliferation. This was possible due to their differentiated impact on the expression of *dur3* and *nrt2* genes and modification of transcription rates in the mixotrophic *P. cordatum*. The results allowed for the conclusion that the knowledge on nitrogen redundancy, diversity and proportions of N-substrates in the environment is of paramount importance. The incorporation of these data into ecological models could increase the precision of the future HAB forecasts under global N pollution and eutrophication.

### 5.2. Total Nitrogen Niche Limit as a Potential Predictor of Dinoflagellate Bloom Formation

Along with different nutrient sources, natural systems are characterized by fluctuating light, episodic nutrient pulses, variable prey communities, and shifting thermal gradients. For these reasons, the kinetics of trophic switching in mixoplankton under the dynamic external conditions and their effects on cell proliferation remain poorly characterized [34]. Additionally, inter- and intra-population heterogeneity of cells, as well as the ambiguity of dinoflagellate behavior under various stresses [79,91] and complexity of their life cycles [39,89,93,95,189,190], hampers the usage of the available data on dinoflagellate cell biology for prognostic mathematical modeling of their HABs. Furthermore, the molecular mechanisms linking environmental sensing to the coordinated regulation of phototrophic and heterotrophic machinery are only just starting to be mapped [34].

Nevertheless, certain advances in unveiling the linkage of some environmental prerequisites and fine cellular–molecular mechanisms underlying dinoflagellate blooms have been achieved in recent decades. For example, it has been shown that the concentration of total nitrogen (TN) in water affects the development and growth of protist cells as well as the overall phytoplankton composition, abundance and distribution; thus, TN can be considered as an anthropogenic stress marker [5,191,192,193,194]. Recently, it has also been demonstrated that dinoflagellates with the widest TN niche limits (along with broad niches for total phosphorus, water temperature and salinity) have the strongest potential for the most frequent bloom events in fluctuating coastal environments [114]. The latter study suggested a conceptual model for merging the bloom-forming capacity of dinoflagellates with the width of their TN niches (Figure 1). This approach allowed for coming closer to prediction of red tides.

### 5.3. The Role of Total Nitrogen in Patterning the Diversity of Dinoflagellate Assemblages

A broad range of organic and inorganic resources used by mixoplankton underpins the coexistence of quite a large number of mixotrophic dinoflagellates in space and time, as demonstrated by the data from the Baltic coastal waters [33,102,113,114]. Recently, we proposed a research hypothesis, suggesting that the coastal regions with TN concentrations that are optimal for bloom-forming dinoflagellates host a greater taxonomic diversity of these protists compared to the areas with non-optimum TN content [42]. However, unexpectedly, this hypothesis was not supported by the analyses of the data on 82 dinoflagellate species in the 44-year-long database from the southwestern Baltic Sea [42]. Instead, surprisingly, the highest species richness of these planktonic protists was observed at a relatively low eutrophication level (TN < 15 µmol/L). Moreover, the study has demonstrated that the diversity of dominant bloom-forming dinoflagellate species decreased gradually from low to moderate, high and very high eutrophication levels. The minimum number of species was observed in extremely eutrophic waters at TN > 45 µmol/L [42]. These results diverge from the theoretically expected species-richness maximum at intermediate TN concentrations, as predicted by the intermediate disturbance hypothesis (IDH). The IDH postulates that maximum species richness should be expected at moderately stressed conditions [195]. A possible though speculative explanation of this discrepancy could involve the linkage between the dinoflagellate growth rates and TN disturbance frequencies rather than magnitude [196,197]. As shown earlier, usually a species diversity maximum is observed when disturbance frequencies are equal to a three-generation cycle [198]. For dinoflagellates at nutrient-saturated (i.e., optimum) conditions, the growth rates between ca. 0.3 and 0.7 day^−1^ are known from laboratory experiments [199], which is in accord with field observations [200] and the knowledge that at suboptimal conditions, the algal growth rates decrease. Consequently, this will shift the optimum disturbance pattern from ca. 4–10 days to much longer periods, allowing for disturbance factors other than TN to serve as stressors affecting the dinoflagellates’ competition pattern. In turn, slower growth of the superior competitor generally favors the enhanced survival time of the inferior ones. In such a case, the relation between these parameters could secure different competition patterns at the optimal and suboptimal TN conditions, promoting higher species richness of mixotrophic dinoflagellates at suboptimal TN concentrations due to lower growth rates and, therefore, less effective competition for nutrients [42].

Thus, similarly to the ambiguous effects of benthic–pelagic coupling involving HAB-forming mixotrophic dinoflagellates [18], the effects of total nitrogen redundancy on dinoflagellate diversity is also nonlinear and enigmatic. This fact demonstrates that nutrient-driven regulation of biodiversity and trophic strategies, including mixotrophy, is a dynamic, multi-faceted stoichiometric optimization. It is shaped by the cell assimilation efficiency, the functional architecture of aquatic assemblages and the complex ever-changing reality of the marine environment [34]. Therefore, the role of TN excess and overall nitrogen redundancy in dinoflagellate cells growth, gene expression, transcriptional responses and population development of these mixoplankters requires further investigations.

### 5.4. Some Perspectives of Nitrogen-Excess Studies

Prospective studies could include testing the applicability of Tilman’s resource competition theory (RCT; [201,202,203]), aiming to unveil the unexpected response of dinoflagellate biomass to nitrogen availability, as described above. Previously, the applicability of RCT has been shown by both theoretical and practical approaches (e.g., [204,205]), at least for the competitive relations between dinoflagellates and diatoms. However, with respect to nitrogen as a co-limiting resource, the competition of diatoms and dinoflagellates with cyanobacteria still needs to be unravelled. The existing knowledge refers to several potential mechanisms ranging from vertical migration (e.g., [206]) to allelopathic interactions (e.g., [207]). However, a comprehensive study, which also takes into account the variable and complex species-specific trophic strategies [204], is still lacking.

## 6. Nitrogen and Phosphorus Deficiency as Major Stressors Modulating Life Cycles of Dinoflagellates in the Batch Culture

### 6.1. The Role of Phosphorus Availability in Dinoflagellate Cell-Cycle and Life-Cycle Modulation

Nitrogen and phosphorus deficiency limits vegetative cell proliferation and can lead to cell-cycle arrest, as shown for various dinoflagellate species in laboratory experiments (e.g., [132,133,140,208,209]). Particularly, phosphorus deficiency leads to cell-cycle arrest in the G1/G0 phase, although the cells continue to grow and increase in size [130,132,133]. At the molecular level, the lack of nutrients suppresses cell-cycle regulators such as G1/S-specific cyclin and cyclin-dependent kinases [122,210]. In *Alexandrium catenella*, phosphorus deficiency reduces the expression of calcium-dependent protein kinase (CPK), which plays a crucial role in signal transduction and cell division in plants. In contrast, fizzy/cell division cycle 20-related protein (FDC) is upregulated; it acts as a negative regulator of the cell cycle, inhibiting cell division by regulating the anaphase-promoting complex (APC) and maintaining the required levels of mitotic proteins [133]. More importantly, nitrogen and phosphorus depletion serves as a notable signal, which initiates life-cycle transitions promoting long-term population survival.

Phosphorus deficiency can trigger the transfer to the sexual phase in complex life cycles of diverse marine dinoflagellates (Supplementary Materials, Table S1). Among those species are *Prorocentrum cordatum*, *Lingulaulax polyedra* (syn. *Lingulodinium polyedra*), *Gymnodinium nolleri*, *Protoceratium reticulatum* and *Alexandrium minutum* [95,211,212,213,214]. These processes included gamete formation and fusion, planozygote formation and encystment.

Life-cycle transition has been well described in the clonal culture of *P. cordatum*, where two weeks of phosphate deprivation led to an increase in the proportion of planozygotes with relative nuclear DNA contents 2C and 4C [95]. Transcriptomic analysis at this stage revealed an increase in the expression of six homologs of meiosis-associated genes—*rad51*, *spo11*, a gene coding for a RAD21/REC8-like protein, *exo1*, *msh4* and *msh5*—which are responsible for meiotic recombination and double-strand break DNA reparation [96]. The addition of a full medium containing sufficient amounts of all the nutrients stimulated cell division and transition to a vegetative 1C phase [95].

### 6.2. The Role of Nitrogen Deficiency in Dinoflagellate Life-Cycle Modulation

Nitrogen limitation also serves as a nutrient-related trigger of transition to the sexual phase (Table S1). In laboratory experiments, cultivation under nitrogen deficiency induced sexuality in various marine dinoflagellates: *Alexandrium taylori*, *Karenia brevis*, *Kryptoperidinium triquetrum* (syn. *K. foliaceum*), *Levanderina fissa* (syn. *Gyrodinium uncatenum*) and *Scrippsiella lachrymosa* [93,215,216,217,218]. In *Lingulaulax polyedra*, planozygotes encysted in a N-depleted medium, like in P-depleted conditions, although nitrogen deficiency yielded more temporary cysts, while phosphorus deficiency produced a greater yield of resting cysts [211]. *Gymnodinium catenatum* showed a similar switch in response to nitrogen deficiency (as well as phosphorus deficiency). However, only this condition, like replete medium, allowed most planozygotes to undergo division. It also enabled more cysts to successfully germinate and retain viability, despite frequent formation of faulty cyst walls [219].

Notably, the available literature on freshwater species mostly reports the effects of nitrogen deficiency on reproductive strategy that are consistent with some of the more complex ideas about nutrient regulation in freshwater ecosystems. Several studies describe *Peridinium* species induced to enter the sexual process, including encystment of planozygotes [220,221,222,223,224] (Table S1).

Transcriptomic data reflecting reproductive strategy transitions in response to nitrogen limitations in dinoflagellates also remain sparsely discussed. Nevertheless, *Alexandrium minutum* has been shown to upregulate some genes coding for meiosis-associated proteins under nitrogen deficiency, for example, homologs of the double-strand break repair protein MRE11, topoisomerase II, DNA mismatch repair protein MLH3 and about two dozen homologs of the RNA-binding protein MEI2 [225]. MEI2-like proteins are abundant in dinoflagellates and appear to form two putative functional sets: one related to the sexual phase and another associated with encystment of vegetative cells (reviewed by [60]). Two *mei2*-like genes also altered their expression in nitrogen-starved cells of *Amphidinium carterae* Hulburt: one was upregulated, and the other slightly downregulated [226].

Simultaneous nitrogen and phosphorus deficiency can also trigger life-cycle transitions, as expected. In some cases, combined nitrogen and phosphorus limitation produces the same effect as single-nutrient limitation. This has been described in *Parvodinium cunningtonii*, *Peridinium cinctum* and *Scrippsiella acuminata* [223,227,228] (Table S1). In other cases, combined deficiency can enhance the impact; for example, gamete fusion and planozygote encystment occurred in a larger proportion in a -P-N medium than under N- or P-only deficient conditions in *Gymnodinium catenatum* [219]. The overview of nutrient deficiency-driven transitions between dinoflagellate life-cycle stages and associated changes in the expression of genes involved in the meiotic process is presented in Figure 2.

Culture aging without directly altering the medium composition also acts as a condition triggering life-cycle switches. Although in this case it is more accurate to consider a combination of factors affecting the cell population, one of them will most likely be the deficiency of nutrients. Gamete pairs were most easily found in older cultures of *Pfiesteria piscicida* Steidinger & J.M.Burkholder [229]. Mating gametes occurred in the late exponential or stationary cultures of *Prorocentrum cordatum*, *Prorocentrum donghaiense* D.Lu and *Karenia mikimotoii* [100,190,230].

### 6.3. The Role of Nitrogen and Phosphorus Availability in Resting Cyst Formation and Germination

Nutrient deficiency can specifically trigger the direct transition from vegetative growth to resting cyst formation, since cysts can be formed not only as a dormant stage in zygote development but also as a survival strategy when conditions for the vegetative phase are unfavorable. Resting cysts are protected against viruses, predators and parasites. Nitrogen limitation effects have been shown in some marine armored and naked dinoflagellates, including *Alexandrium minutum* (syn. *A. lusitanicum*), *A. taylori*, *Gymnodinium corollarium*, *Lingulaulax polyedra* and *Scrippsiella acuminata* (syn. *S. trochoidea*) [93,208,228,231]. A slight effect was reported for the peridinialean *Ensiculifera* sp. [208]. *Karenia mikimotoi* forms cysts referred to as temporary; however, the presented micrographs do not allow for confident identification of their type [209]. In freshwater species, nutrient depletion can also stimulate encystment [224]. In P-depleted conditions, encystment has been shown in *Alexandrium taylori*, *Lingulaulax polyedra* and *Gymnodinium catenatum*. In the latter two species, phosphorus depletion has a stronger effect on cyst production than nitrogen limitation does [93,208,219], while in *Scrippsiella acuminata* and *Alexandrium minutum*, the opposite was true [208,228]. Thus, the nutrient deficiency effects cannot be generalized across taxa. Yue et al. [228] also showed that the life-cycle transition was consistently accompanied by the altered expression of cell-cycle regulators, including cyclin B and cyclin-dependent kinase 1; expression of these genes in resting cysts remained moderate but significantly lower than that in fast-growing vegetative cells.

It is noteworthy that cysts must possess sufficient ATP reserves to germinate. For example, *S. acuminata* cysts can take up phosphate from the environment [232]. This enables dinoflagellate cysts to accumulate phosphorus from benthic sources rich in Pi for subsequent use during germination and ensuing growth.

Sexual resting cysts (hypnozygotes) undergo meiosis during germination. Deng et al. [233] performed transcriptomic analysis of mature cysts sampled from 50–60-day-old cultures of *S. acuminata*. Increased expression of genes potentially involved in meiosis—*dmc1*, *spo11* and some genes encoding MEI2-like proteins—was indicated [233]. At the same time, several *mei2*-like genes were downregulated. However, the actual life-cycle status of the resting cysts in this study—vegetative or sexual—has not been entirely clear [233]. A more recent study demonstrated overexpression of a similar set of genes, namely *spo11*, *dmc1* and also *hop2*, *mnd1*, *msh4* and numerous *mei2* homologs, during the germination of *S. acuminata* hypnozygotes formed in a culture system, i.e., during the process when meiosis reliably occurs [97].

### 6.4. Beyond Nutrient Limitation: Multiple Triggers and Laboratory Constraints

Transition in the life cycle is a very complex process, which is regulated not only by simple nutrient deprivation but also by multiple physical and chemical factors, such as salinity, temperature and light intensity; this effect was well demonstrated for *Alexandrium minutum* [213,234]. Decreased salinity and increased temperature together with phosphorus deprivation promoted planozygote formation and subsequent resting cyst production. In addition, a specific irradiance level was shown to be an important factor promoting resting cyst production together with nutrient deficiency. For *A. minutum*, the optimal light intensity was 150 μE m^−2^ s^−1^. Higher irradiances reduced the total encystment yield [234]. The low nitrogen in combination with high irradiance had a significant impact on *Peridinium cinctum* [224]. Specifically, in a *P. cinctum* culture, only the highest temperature tested (22 °C) induced a higher cyst yield under phosphorus deprivation compared with the control (full medium) [227]. Cell density is another important factor, which increases the probability of gamete mating. Moreover, the presence of appropriate genetically diverse mating partners is a crucial factor for heterothallic species [213,234].

Despite the growing number of transcriptomic observations, mechanistic evidence for nutrient-driven life-cycle regulation remains limited and speculative. The available literature indicates that the cells respond to their intracellular N and P status rather than directly to external nutrient concentrations. In *Levanderina fissa* (syn. *Gyrodinium uncatenum*) and *Alexandrium tamarense*, sexual reproduction and encystment began when intracellular N or P pools approached a minimum cellular quota [218,235]. In a *P. cordatum* life-cycle study, the transition to the sexual phase appeared only after one week, when the phosphate concentration in the medium had dropped below the detectable level [95]. Thus, external nutrient depletion may first reduce the intracellular N or P quota below a species-specific threshold, which is then detected by nutrient-sensing pathways. The molecular sensor of N status remains unknown, although changes in cellular N balance and N assimilation metabolites are plausible signals. For phosphorus, SPX-domain-containing proteins are promising candidates because they regulate phosphate homeostasis in other photosynthetic eukaryotes such as diatom *Phaeodactylum tricornutum* and have been identified among P-responsive genes in *P. cordatum* [95,96,236]. Nutrient sensing may then suppress vegetative cell-cycle activity and activate sexual differentiation and/or encystment. Consistent with this model, N deficiency induces cyst formation in *Scrippsiella acuminata*, with changes in cyclin B and CDK1 expression observed in this dinoflagellate [228].

Thus, nutrient deficiency indeed affects dinoflagellate life-cycle transitions, but these effects cannot be fully generalized across the taxa e.g., [237,238,239,240,241,242,243]. Life cycles have been described for a very limited number of ecologically important dinoflagellate species. Nevertheless, based on the available research data, several patterns can be mentioned. A lack of nitrogen appears to be the more common and effective trigger of the transition from vegetative growth to sexual reproduction and/or encystment across the class Dinophyciae, particularly, in members of the orders Peridiniales and Gymnodiniales. However, in some taxa, phosphorus limitation appears to play a more prominent role, for example, in Gonyaulacales, especially in the well-studied, toxin-producing genus *Alexandrium*. However, even in this example, nitrogen availability can have a pronounced effect on the life cycle, highlighting species-specific variation in nutrient control of sexuality and encystment (Table S1). Moreover, it should be kept in mind that all the effects described in the current section were demonstrated in laboratory cultures under strictly controlled experimental conditions. Consequently, these cell responses should be interpreted cautiously when extrapolating to natural populations that can be influenced by numerous physical and chemical factors, as well as by cell density, the availability of genetically diverse mating partners, the presence of prey or predators and the surrounding bacterial community. Experimental research combined with field studies, in particular field transcriptomics, will help unravel the actual molecular mechanisms underlying dinoflagellate life-cycle regulation.

## 7. Field Transcriptomic Evidence of Dinoflagellate Life-Cycle Switching

Field transcriptomics, combined with microscopic observations of cells, provide direct though still taxonomically specific evidence that dinoflagellate life-cycle transitions leave a measurable gene-expression footprint in natural populations (Table 2). In part, these footprints can be primarily used to detect and predict bloom development. Increased expression of genes associated with meiosis, such as *dmc1*, *spo11*, *mnd1*, *msh6*, *rad21*, *rad51*, *exo1*, *mns1*, *smc3*, *smc2* and *smc1*, was demonstrated in metatranscriptome analysis of samples taken from natural populations of *Prorocentrum shikokuense* during a bloom [97]. In a *Karenia mikimotoi* bloom, the genes *rad54*, *msh2*, *rad21*, *spo11*, *dmc1*, *mnd1* and *smc3* were upregulated. This observation became the basis of the “sex-for-proliferation” hypothesis, suggesting that sexual reproduction can facilitate population expansion or bloom maintenance without encystment [97]. Although this hypothesis relied mostly on metatranscriptomics data, it was also underpinned by the results of cytometric analysis of *P. shikokuense* and *K. mikimotoi* cells from bloom samples, which confirmed alterations in DNA content [97]. Moreover, a recent study validated the “sex-for-proliferation” hypothesis by an automated microscopy survey in natural bloom and experimental conditions [244]. The authors showed an increase in gamete fusion and rapid cell reproduction via meiotic divisions without encystment during a *Dinophysis acuminata* bloom peak [244]. Nevertheless, direct cytological evidence supporting this concept under field conditions remains scarce and requires further in-depth study by the “integration of physiological measurements, microscopic observations, and molecular analyses” [97].

Yu et al. [245] argued that bloom development and collapse of *Karenia longicanalis* Z.B.Yang, I.J.Hodgkiss & Gert Hansen were governed by multiple processes, especially energy and nutrient acquisition, on the one hand, and grazing, microbial attack and community turnover, on the other. They also identified 270 bloom-associated genes, which included cell proliferation genes comprising those regulating sexual reproduction and cell division cycle [245]. This shows the environmental context in which reproductive switching becomes visible: these are not isolated expression events in culture but parts of a whole-community bloom trajectory. In addition, three years of metatranscriptome analysis of *Alexandrium minutum* blooms showed that the species displayed a very specific expression pattern during natural blooms, with lower expression of photosynthesis and central-metabolism transcripts than the surrounding community [247]. Moreover, despite the correlation of expression patterns with the concentration of nutrients (phosphorus, nitrogen and silicate), genes coding for proteins involved in nutrient uptake and metabolism did not display extensive alterations [247].

Deng et al. [246] used a dinoflagellate-specific DinoSL mRNA “hook” to analyze sediment-buried cyst assemblages and found that most major metabolic and regulatory pathways remained transcriptionally active, except photosynthesis; they also implicated autophagy and abscisic acid/gibberellin signaling in cyst dormancy and germination. This makes the sediment bank look less like a silent reservoir and more like a transcriptionally regulated dormant stage. Related sediment studies found HSP90 and small heat-shock-protein transcripts in field-collected or buried cysts [248].

Culture-based studies make the field patterns easier to interpret because they identify the transcript modules that track encystment and dormancy. In *Scrippsiella acuminata*, encystment and cyst dormancy were associated with strong reprogramming of photosynthesis, active catabolism, meiotic recombination genes, *mei2*-like genes and abscisic acid metabolism [233]. In the latter study, it has been demonstrated that energy storage dominated the immature-cyst stage, energy consumption dominated germination, and “genetic information processing” marked *S. acuminata* resting cysts [249]. In *Prorocentrum cordatum*, cold-induced temporary cysts showed repression of calcium and protein kinase signaling, and some specific RNA-binding proteins, particularly proteins containing RNA-binding pentatricopeptide repeats and several MEI2-like proteins that were determined as relevant to non-sexual cyst formation [92]. These data still need to be summarized and processed to identify genes that could serve as molecular cues of processes occurring in natural populations. Such attempts have been repeatedly made for a long time. For example, Coyne and Cary [250] showed that specific cyst transcripts could be detected both in laboratory cultures and directly in natural estuarine sediments by RT-PCR, supporting mRNA as a proxy for viable cysts. However, this challenging research field requires further expansion, focusing on the search for molecular signatures of shifts in cell development in natural dinoflagellate populations that can be incorporated in future mathematical models aimed at HAB forecasting.

## 8. Outlook: Molecular Biology Tools and Ecological Modeling Secure the Progress of Translational Aquatic Ecology

### 8.1. Main Conclusions

The expansion of HABs as a consequence of the enhanced inflow of anthropogenic nitrogen and phosphorus to aquatic ecosystems is one of the most crucial environmental problems nowadays [6,8,11,14,17,42,153,191]. Extensive studies of dinoflagellate blooms over the past decades have allowed for the identification of several important milestones on the way toward predicting HABs (Figure 3). These steps include the following:

- The development of mathematical modeling based on modern concepts, theories and the knowledge of molecular characteristics of dinoflagellates (e.g., expression of specific genes regulating cell-cycle shifts and nutrient acquisition modes);
- Experimental evaluation of cellular features and processes responsible for proliferation and population dynamics (e.g., kleptoplasty efficiency, ability to form cysts and specific cyst dynamics);
- The assessment of major autecological characteristics (e.g., intra-population heterogeneity, autotrophy/heterotrophy balance, ecological niche sizes, etc.) of common HAB-forming dinoflagellate species.

The consideration of molecular mechanisms regulating life cycles, cysts production and excystment in these protists is also essential for the development of basic ecological theories and practices. Specifically, the theoretical basics of the classical intermediate disturbance hypothesis [195] and the resource competition theory [201] form an indispensable background for environmental modeling. Similarly, newly suggested concepts like the protistan species maximum for the zone of critical salinity 5–8 [251] and the mixoplankton concept [148] are of particular importance for understanding HABs. In addition to the regular consideration of the intracellular response to nutrient deficiency, it is crucial to take into account the possibilities of the minimum cellular quota and the presence of potential internal molecular sensors for nutritional status [218,235]. The hypothesis describing the “sex-for-proliferation” scenario (as an alternative to the “sex-for-encystment” concept) proposed by Lin and co-authors [97] likewise expands the understanding of bloom development strategies. Genomics, transcriptomics and molecular phylogeny data can substantially contribute to the progress of chaos theory [1,3,4] and secure the improvement of translational aquatic ecology in general [37,38]. Coupling the achievements of these theoretical and experimental studies with omics data and mathematical modeling is a basic prerequisite for the adequate comprehension and interpretation of the complex, nonlinear biotic interactions within aquatic communities and ecosystems.

### 8.2. Limitations and Bottlenecks

Until recently, molecular studies of bloom-forming microorganisms and ecological research of the reasons and triggers, onset conditions, drivers, causes of decay and ecological consequences of algal blooms have been progressing largely in parallel. Although different studies aimed at resolving similar tasks like the assessment of possible consequences of HABs, little or no interference in their design, research strategies and methodologies can be considered as a major limitation of such a discrete approach. Now it has become possible to attempt merging the approaches of different disciplines within a framework of translational aquatic ecology, which implies close linkage of cellular biology, molecular ecology and omics with the classical ecological theories and environmental protection practices. This integrated approach can help avoid facing current technical bottlenecks such as in situ quantification of kleptoplasty efficiency, in situ assignment of life-cycle stages from metatranscriptomes, the gap between transcript signal and real phenotype and challenges in multi-factor experimental design. Additionally, some essential experimental cellular and autecological data are still insufficient. Particularly, such basic prerequisites of HAB modeling like kleptoplasty efficiency, intra-population heterogeneity of dinoflagellates, their species-specific autotrophy/heterotrophy balance and cyst dynamics suffer from scarce empirical data.

### 8.3. Future Research and Model Integration

Resolving the abovementioned limitations by obtaining additional empirical data and their smooth integration with conceptual and mathematical modeling is a challenging task and a priority research direction for the prospective HAB investigations and forecasts. Meanwhile, current modeling spans multiple levels of abstraction [33,34,252,253,254]. Trait-based models predict elevated trophic energy transfer efficiency, larger average organism size and altered vertical carbon flux when mixotrophy is properly parameterized [186,255]. Empirical habitat models can forecast bloom probability of dinoflagellates from certain physical and biogeochemical predictors [253]. The recent kleptoplasty model proposes the criterion for quantifying the contribution of plastids, retained by dinoflagellates during mixotrophy, to their photosynthesis, cell growth and the autotrophy/heterotrophy balance [33].

The latter example clearly demonstrates how a new ecological indicator, the kleptoplasty index, was developed as an integrated result of conceptual and mathematical modeling, laboratory investigations of dinoflagellate physiology and theoretical experiments. It was the coupling of these approaches that allowed for assessing the quantitative contribution of kleptoplasty to overall photosynthesis, cell proliferation and, finally, population growth and consequent HAB formation. Thus, application of the TAE strategy by integrating recent achievements in research and mathematical modeling can help produce realistic forecasts in the future and come closer to combatting the devastating effects of HABs.

Moreover, it appears now that field transcriptomics likely provides direct evidence that dinoflagellate life-cycle transitions leave measurable gene-expression footprints in natural populations. These footprints, in turn, can be used in mathematical models to detect and predict the enhanced protist population growth and development leading to HABs.

Among the molecular signatures that can be proposed for inclusion in such models are the expression of genes involved in cell-cycle progression (for example, genes coding for cyclins) and activity of a sufficient set of meiosis-associated genes (first and foremost, specific genes responsible for double-strand break formation and processing and for crossover events). Additionally, these could be genes coding for proteins participating in transport processes and metabolic pathways, including hormone metabolism (the necessary sets should be developed for the certain cases), and proteins associated with general physiological stress.

The data reviewed in this article clearly demonstrate that the activities of selected sets of genes could be reliable signatures of cellular status and should be tested for their usage in modeling. Future development of mathematical models considering the above molecular variables as feasible model parameters can provide the opportunity to merge the newest outcomes of different disciplines: molecular biology and omics, protist cell morphology and physiology and field and laboratory experimental aquatic ecology. This integration will secure a promising perspective for the progress of these mutually interlinked topical research directions.

## Figures and Tables

**Figure 1:**
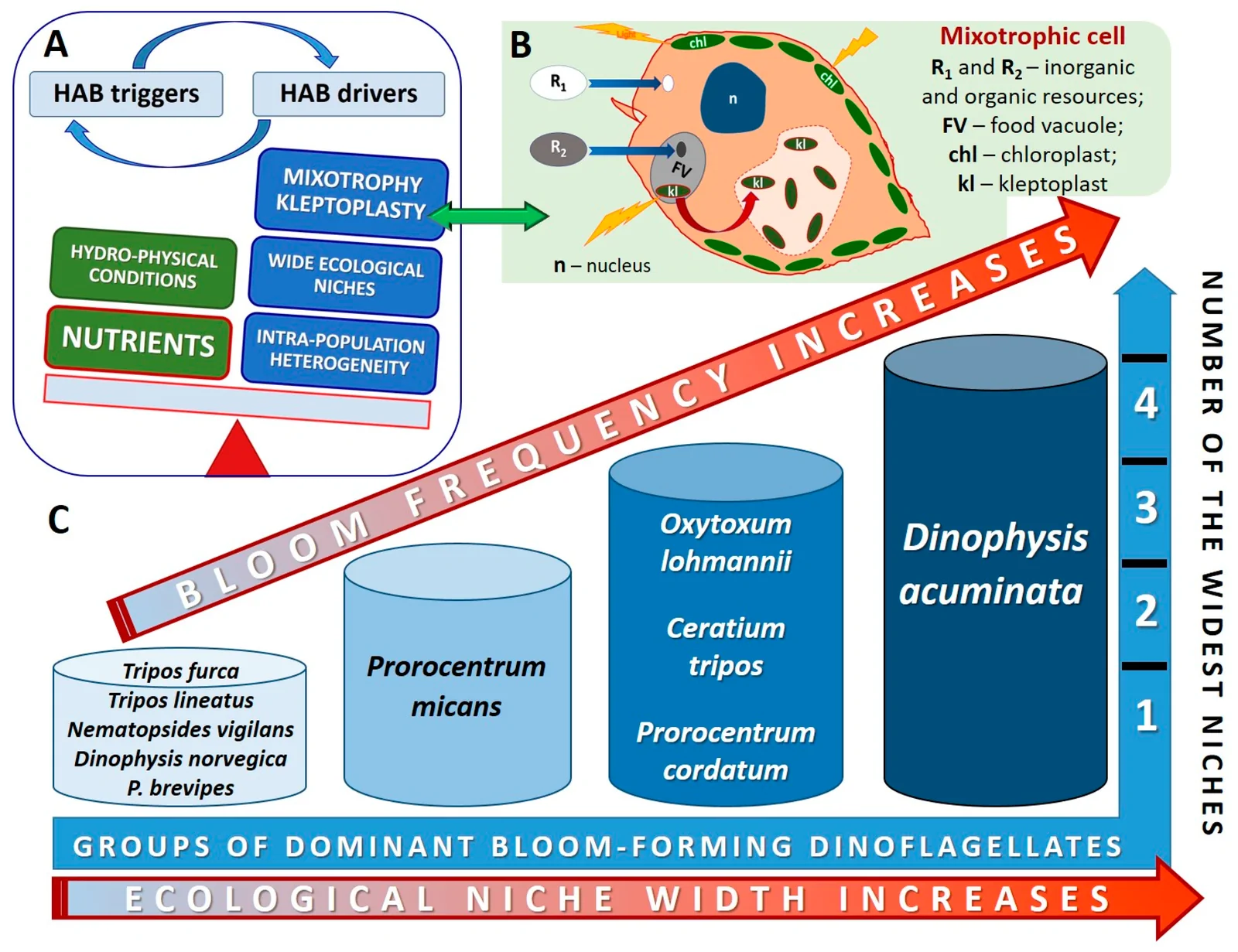
Schematic representation of the established data on the inter-relations between bloom frequency of mixotrophic dinoflagellates and their ecological niche sizes, basic external triggers and major internal HAB drivers. (**A**) Schematic illustration of the crucial role of mixotrophy and kleptoplasty of dinoflagellates in the interplay of nutrient content and abiotic conditions in the environment as external triggers, on the one hand, and cellular characteristics as internal drivers of HABs, on the other hand (based on the data from [18]). (**B**) Scheme of a conventional photo-phago-mixotrophic dinoflagellate cell structure (modified considerably from [19]). (**C**) Schematic representation of the data on bloom frequency dependence on the ecological niche width of the dominant dinoflagellate species in the Baltic Sea. Dinoflagellates: *Tripos furca* (Ehrenberg) F.Gómez (syn. *Ceratium furca*), *Tripos lineatus* (Ehrenberg) F.Gómez (syn. *Ceratium lineatum*), *Nematopsides vigilans* (S.M.Marshall) Greuet (syn. *Proterythropsis vigilans*), *Dinophysis norvegica* Claparède & Lachmann, *P. brevipes—Protoperidinium brevipes* (Paulsen) Balech, *Prorocentrum micans* Ehrenberg and *Oxytoxum lohmannii* Tillmann & Gottschling (syn. *Amphidinium crassum*). The species are grouped according to the niche limits for water temperature, salinity, total nitrogen and total phosphorus concentrations (based on the results of the Principal Component Analysis of 4534 datasets collected in 1972–2016, from [114]).

**Figure 2:**
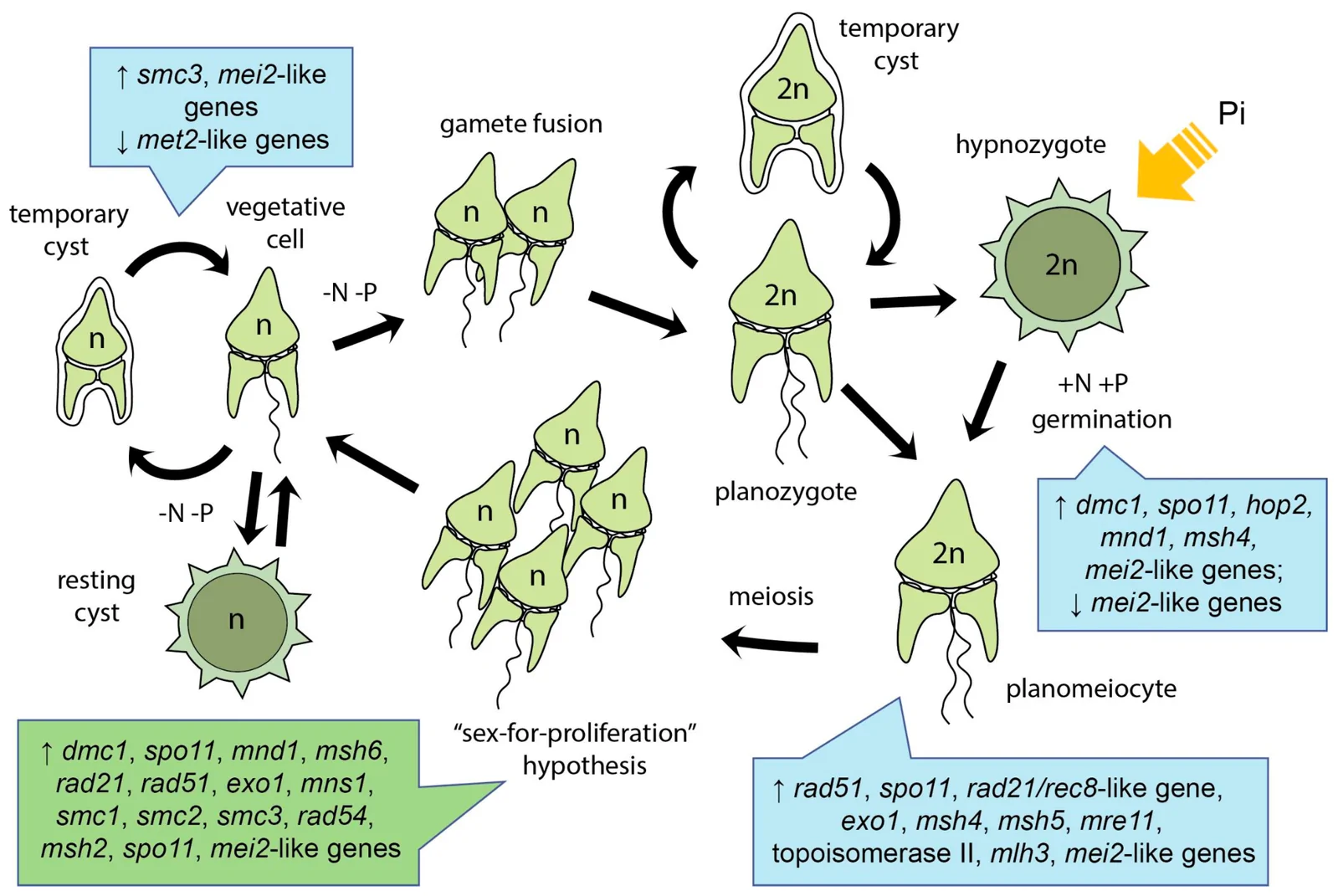
Schematic representation of nutrient deficiency-induced transitions between dinoflagellate life-cycle stages and associated changes in the expression of meiosis- and recombination-associated genes, including meiosis-associated and encystment-associated *mei2*-like genes. Nitrogen and/or phosphorus deficiency (-N and -P) can promote the formation of temporary or resting cysts or induce the transition from vegetative cells toward gamete formation and fusion, followed by planozygote and hypnozygote formation. Under nutrient-replete conditions (+N and +P), hypnozygotes germinate and give rise to planomeiocytes, which undergo meiotic division to produce haploid cells. Changes in the expression of selected genes are indicated by upward (↑) or downward (↓) arrows. Blue boxes represent transcriptomic data obtained in laboratory cultures; the green box shows transcriptomic data from natural blooms.

**Figure 3:**
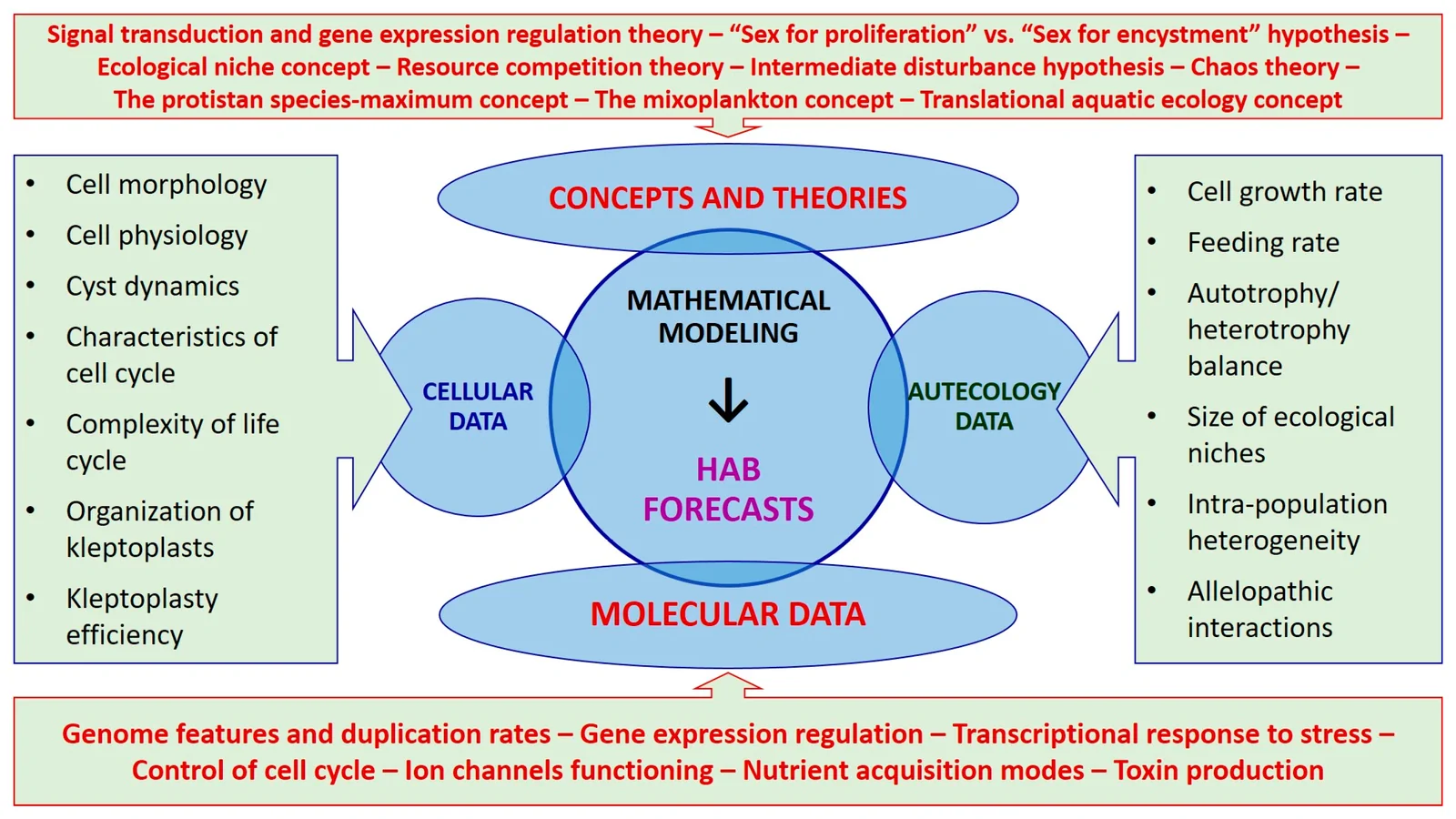
Basic milestones (development of concepts and theories, research of species autecology and molecular and cellular characteristics) on the way toward forecasting red tides of mixotrophic dinoflagellates. Experimental cellular and autecology data (left and right boxes) are still insufficient; particularly the modules “kleptoplasty efficiency”, “intra-population heterogeneity”, “autotrophy/heterotrophy balance” and “cyst dynamics”, as basic prerequisites of HAB modeling suffer from scarce empirical data.

**Table 1:** Statistically significant (*p* < 0.05) correlations between the abundances (cells/mL) of dinoflagellates *Prorocentrum cordatum* and their prey, the cryptophyte microalgae *Hemiselmis virescens*, in the southwestern Baltic coastal waters, with outliers included (outlier: ALARM) and excluded (outlier: 0). The critical “outlier level” was calculated as “3 × SD + mean”. Abbreviations: SD—standard deviation, m—slope, SE– standard error, F—the F-test of overall significance, and n—number of analyzed datasets. The analysis demonstrates the co-existence of dinoflagellates *P. cordatum* and *P. micans* with their prey cryptophyte microalgae by revealing strong, statistically significant positive correlations between their abundances. Based on the data from the 44-year-long (1972–2016) phytoplankton database described earlier [102,113,185].

	Outliers Included		Outliers Excluded	
Species	*Prorocentrum cordatum*	*Hemiselmis virescens*	*P. cordatum*	*H. virescens*
Outlier	ALARM	ALARM	0	0
Max abundance	1497	2453	323	1286
Mean abundance	108.59	328.01	21.81	195.20
SD	366.17	668.43	80.45	395.90
Outlier level	1207.09	2333.32	-	-
m		0.478		0.120
SE		0.069		0.044
R^2^		0.76		0.35
F	target 4.45	47.92	target 4.49	7.56
n	17	17	16	16

**Table 2:** Evidence for life-cycle transitions in natural dinoflagellate populations. Abbreviations: BS—bloom population samples, FS—field-collected sediment samples, IFC—imaging-in-flow cytometry, and MT—metatranscriptomics.

Species	Sample Type	Methods	Expression Signal/Observation	Life-Cycle and Bloom-Development Interpretation	Reference
*Prorocentrum shikokuense*	BS	MT	Increased expression of *dmc1*, *spo11*, *mnd1*, *msh6*, *rad21*, *rad51*, *exo1*, *mns1*, *smc3*, *smc2* and *smc1*	Sex associated with bloom proliferation	[97]
*Karenia mikimotoi*	BS	MT	Increased expression of *rad54*, *msh2*, *rad21*, *spo11*, *dmc1*, *mnd1* and *smc3*	Sex associated with bloom proliferation	[97]
*Dinophysis acuminata*	BS	IFC	Increased gamete fusion and fast meiotic divisions without encystment	Sex bypasses the vegetative cell cycle and drives boom development	[244]
*Karenia longicanalis*	BS	MT	Increased expression of *msh2*, genes encoding “cell-division cycle proteins”	Asexual reproduction associated with bloom formation	[245]
Dinoflagellate cyst assemblages	FS	MT, hormone treatment	Activity of metabolic and regulatory pathways; suppression of photosynthesis; promotion of germination under gibberellin treatment	Metabolic activity during dormancy; phytohormone role in modulating dormancy/germination of resting cysts	[246]

## Data Availability

No new data were created or analyzed in this study. Data sharing is not applicable to this article.

## Supplementary Materials

The following supporting information can be downloaded at: https://www.mdpi.com/article/10.3390/ijms27198622/s1.
