# Supplementary material for: Molecular Insights into Nutrient-Driven Regulation of Trophic Strategies and Life-Cycle Shifts in Bloom-Forming Mixotrophic Dinoflagellates
Source: Int J Mol Sci. 2026 Sep 26;27(19):8622. doi: 10.3390/ijms27198622 (PMC13665112; doi:10.3390/ijms27198622)
Supplement: Supplementary file 1 [file ijms-27-08622-s001.zip › ijms-4551393-supplementary.pdf]

Supplementary Materials

Review article:
"**Molecular Insights into Nutrient-Driven Regulation of Trophic Strategies and Life-Cycle Shifts in Bloom-Forming Mixotrophic Dinoflagellates**" by Sergei O. Skarlato, Mariia A. Berdieva, Vera O. Kalinina, Hendrik Schubert, Irena V. Telesh

Table S1. The effects of phosphorus deficiency (-P) and/or nitrogen deficiency (-N) on the life cycles of various dinoflagellate species.

Species	Order	Environment	-P	-N	-P-N	References*	Comments**
Alexandrium minutum Halim (synonym *Alexandrium lusitanicum* Balech)	Gonyaulacales	Marine	Induces gamete and planozygote formation, and encystment	Induces encystment[v]	–	[208, 213, 237]	Heterothallism; -N medium gives the highest cyst yield
Alexandrium ostenfeldii (Paulsen) Balech & Tangen	Gonyaulacales	Marine	Ensures relatively low resting cyst[v] yield	–	Ensures relatively high level of resting cyst[v] production	[238]	Heterothallism
Alexandrium tamarense (Lebour) Balech	Gonyaulacales	Marine	Induces sexuality, planozygote formation and encystment	–	Induces encystment[v] (resting cysts formation)	[239, 240]	Heterothallism
Alexandrium tamutum Montresor, Beran & John	Gonyaulacales	Marine	Ensures formation of planozygotes, but they cannot divide and encyst	–	Induces encystment[v]	[237]	Heterothallism
Alexandrium taylori Balech	Gonyaulacales	Marine	Induces resting cysts[v] production	Induces resting cyst[v] production	–	[93]	-N medium induces long-term encystment, -P medium is a more effective condition for short-term cysts formation
			Ensures planozygote	Induces sexuality, gamete paring and			

			encystment (short-term cysts formation)	fusion, planozygote encystment (resting cysts formation, sometimes short-term cysts formation)			
Biecheleria halophila (Biecheler) Moestrup, Lindberg & Daugbjerg (syn. *Woloszynskia halophila* Elbrächter & Kremp)	Suessiales	Marine	–	Ensures cell size reduction (30-40 days), some cysts[v] formation	–	[231]	—
Ensiculifera sp. Balech	Peridiniales	Marine	–	Induces encystment[v] (slight effect)	–	[208]	—
Gymnodinium catenatum Graham	Gymnodiniales	Marine	Induces encystment[v]	–	Induces encystment[v] (resting and temporary cysts)	[219, 241]	Heterothallism; gamete fusion and planozygote encystment occur in larger proportion in -P-N medium, zygote division is observed in -N and replete medium, cyst germination is lower in -P medium; -P medium gives significantly higher resting cyst yield at the vegetative phase
			induction of sexuality, gamete formation and fusion, zygote encystment	Induces sexuality, gamete formation and fusion, zygote encystment, zygote division; deficient development of the cyst wall	Induces sexuality, gamete formation and fusion, zygote encystment; induces planozygote formation (this stage persists for up to two weeks before encystment)	[219, 241]	
Gymnodinium corollarium Sundström, Kremp & Daugbjerg	Gymnodiniales	Marine	–	Induces resting cysts[v] formation	–	[231]	—

Gymnodinium nolleri Ellegaard & Moestrup	Gymnodiniales	Marine	Induces sexuality, gamete formation and fusion, zygote encystment	–	–	[212]	—
Karenia brevis (Davis) Gert Hansen & Moestrup	Gymnodiniales	Marine	–	At 15 °C ensures-pellicle cyst[v] formation<br>At 20 °C and 25 °C induces sexuality (mating behaviour), gamete formation and fusion, planozygote formation and encystment (pellicle “possible” cysts)	–	[215]	Heterothallism; in replete medium at 25 °C, induction of sexuality also occurs
Karenia mikimotoi (Miyake & Kominami ex Oda) Gert Hansen & Moestrup	Gymnodiniales	Marine	–	Ensures temporary cysts[v] formation	–	[209]	As the culture time increases, cyst production rate decreases
Kryptoperidinium triquetrum (Ehrenberg) Tillmann, Gottschling, Elbrächter, Kusber & Hoppenrath (syn. *Kryptoperidinium foliaceum* (Stein) Lindemann)	Peridiniales	Marine	–	Induces sexuality, gamete fusion, planozygote formation and encystment	–	[217]	Homothallism
Levanderina fissa (Levander) Moestrup, Hakanen, Gert Hansen, Daugbjerg & Ellegaard	Gymnodiniales	Marine	Induces planozygote formation followed by encystment	Induces planozygote formation followed by encystment	–	[218]	—

(syn. *Gyrodinium uncatenum* Hulburt)							
Lingulaulax polyedra (Stein) Head, Mertens & Fensome (syn. *Lingulodinium polyedra* (Stein) Dodge)	Gonyaulacales	Marine	Induces sexuality, gamete paring and fusion, zygotes encystment	Induces encystment[v]<br>Zygotes encystment	–	[208]<br>[211]	-N medium gives significantly lower resting cyst yield, but higher ecdysal cyst yield
Parvodinium cunningtonii (Lemmermann) Pandeirada, Craveiro, Daugbjerg, Moestrup & Calado (syn. *Peridinium cunningtonii* (Lemmermann) Lemmermann)	Peridiniales	Freshwater	–	Induces sexuality, sexual encystment	Induces sexuality, sexual encystment	[223]	-P treatment causes a less pronounced effect
Parvodinium inconspicuum (Lemmermann) Carty (syn. *Peridinium inconspicuum* Lemmermann)	Peridiniales	Freshwater	–	Induces sexuality, planozygote formation and encystment	–	[222]	—
Peridinium cinctum (Müller) Ehrenberg	Peridiniales	Freshwater	Induces encystment[v] (resting cysts formation)	Induces encystment[v] (resting cysts formation)	Induces encystment[v] (resting cysts formation)	[224, 227]	High irradiance (350 µmol photons) + low N treatment were used; temperature regime affects cyst yield
Peridinium cinctum f. *ovoplanum* Lindemann	Peridiniales	Freshwater	–	Induces sexuality, planozygote formation and encystment, meiotic division	–	[220]	Homothallism

Peridinium gatunense Nygaard	Peridiniales	Freshwater	–	Induces sexuality, planozygote formation and encystment	–	[221]	—
Peridinium willei Huitfeldt-Kaas	Peridiniales	Freshwater	–	Ensures encystment[v] (resting cysts formation)	–	[224]	Homothallism
				Induces sexuality, planozygote formation and encystment, meiotic division		[220]	
Prorocentrum cordatum (Ostenfeld) Dodge	Prorocentrales	Marine	Induces planozygote formation	–	–	[95, 96]	Homothallism, planozygote division occurs after nutrient supplementation
Protoceratium reticulatum (Claparède & Lachmann) Bütschli	Gonyaulacales	Marine	Induces planozygote formation; 6.1% of crosses underwent encystment	–	–	[214]	Homothallism for planozygote formation and division, but only crosses of different isolates can produce resting cysts
*Scrippsiella acuminata (*Ehrenberg) Kretschmann, Elbrächter, Zinssmeister, S.Soehner, Kirsch, Kusber & Gottschling *(*syn. *Scrippsiella trochoidea* (Stein) Loeblich)	Thoracosphaerales	Marine	Induces encystment[v]	Induces encystment[v]	Induces encystment[v]	[228, 232, 242]	-P medium provides the lowest cyst yield

Scrippsiella lachrymosa Lewis ex Head	Thoracosphaerales	Marine	Ensures lower encystment percentage and lower haploid cell yield	Ensures lower encystment percentage and lower haploid cell yield;<br>Induces sexuality, gamete formation, planozygote formation and encystment (resting cysts)	–	[216, 243]	—

* All citations mentioned in the Supplementary Table S1 are included in the main text of the article and the list of References.
** The authors' comments are based on the analysis of published data.
[V] Encystment at the vegetative phase of the life cycle.
"–", no data; "—", no comments.
